# Individual and Population Comparisons of Surgery and Radiotherapy Outcomes in Prostate Cancer Using Bayesian Multistate Models

**DOI:** 10.1001/jamanetworkopen.2018.7765

**Published:** 2019-02-01

**Authors:** Lauren J. Beesley, Todd M. Morgan, Daniel E. Spratt, Udit Singhal, Felix Y. Feng, Allison Cullen Furgal, William C. Jackson, Stephanie Daignault, Jeremy M. G. Taylor

**Affiliations:** 1 Department of Biostatistics, University of Michigan, Ann Arbor; 2 Department of Urology, University of Michigan, Ann Arbor; 3 Department of Radiation Oncology, University of Michigan, Ann Arbor; 4 Department of Radiation Oncology, University of California, San Francisco

## Abstract

**Importance:**

Whether surgery or radiotherapy is the preferred treatment for patients with localized prostate cancer continues to be debated, and randomized clinical trials cannot yet fully address this question. Furthermore, there may be heterogeneity in responses, and the optimal treatment for a patient will depend on his clinical and tumor characteristics.

**Objectives:**

To use a unified statistical approach to compare the association of surgery and radiotherapy with both metastatic clinical failure (CF) and survival in localized prostate cancer and to develop an online calculator for individualized, treatment-specific outcome prediction.

**Design, Setting, and Participants:**

Cohort study for statistical analysis and development of individualized predictions using Bayesian multistate models that jointly consider both CF and survival and adjust for confounding factors. This study used data from patients treated at the University of Michigan between January 1, 1996, and July 1, 2013, with detailed information on treatment, patient and tumor characteristics, and outcomes. Primary analyses were performed in 2017 and 2018. Participants were a cohort of 4544 patients with localized prostate cancer undergoing primary treatment.

**Exposures:**

Radical prostatectomy and external beam radiotherapy.

**Main Outcomes and Measures:**

The clinical outcomes were metastatic CF, death after CF, and death from other causes. The adjustment factors were age, prostate gland volume, prostate-specific antigen level, comorbidities, Gleason score, perineural invasion, cT category, race, and treatment year. An online calculator was developed to estimate risks for multiple outcomes for any patient based on 2 treatment choices and on his clinical and tumor characteristics.

**Results:**

Among 4544 men (mean [SD] age, 61.2 [8.0] years), 3769 underwent radical prostatectomy, 775 received external beam radiotherapy, 157 (3.5%) had CF, 90 (2.0%) died after CF, and 378 (8.3%) died of other causes. Across all patients, there was no significant difference in risk of CF for surgery vs radiotherapy (hazard ratio, 0.80; 95% CI, 0.52-1.23). However, using multistate models, in some cases individualized predictions resulted in different expected outcomes between surgery and radiotherapy for a given patient.

**Conclusions and Relevance:**

In this study, after adjustment for measured confounders, the hazard of CF was similar between treatments on average. However, these data indicate a greater oncologic benefit for some individual patients if treated with surgery and for other patients if treated with radiotherapy. Individualized predictions provide a novel approach to facilitate treatment decision making.

## Key Points

**Question:**

Is surgery or radiotherapy preferred on average for treatment of localized prostate cancer when considering both metastatic clinical failure and overall survival, and is either treatment preferred for a particular patient based on his clinical and tumor characteristics?

**Findings:**

In this cohort study using a Bayesian multistate model fit to data from 4544 patients, no clear difference was found in the hazard of clinical failure between surgery and radiotherapy on average. Additional modeling explores personalized risk for multiple possible outcomes based on the treatment and on clinical and tumor characteristics.

**Meaning:**

The online calculator presents multiple outcomes and can serve as a platform to inform treatment selection in men with localized prostate cancer.

## Teaser

This cohort study uses Bayesian multistate models for a unified statistical approach to compare the association of surgery and radiotherapy with both metastatic clinical failure and survival in men with localized prostate cancer and develops an online calculator for individualized treatment-specific outcome prediction.

## Introduction

The relative merits of using surgery or radiotherapy as the primary therapy for localized prostate cancer have been debated for decades. Large randomized clinical trials comparing surgery vs radiotherapy would provide the highest level of evidence regarding this comparison. The results from 3 contemporary clinical trials are available, 2 of which have fewer than 100 patients.^1,2^ The only large trial is the Prostate Testing for Cancer and Treatment (ProtecT) study,^3^ which randomized 1643 patients to surgery, radiotherapy, or active monitoring. Between the surgery and radiotherapy arms, almost identical rates of prostate cancer deaths (5 of 553 vs 4 of 545) and metastatic events (13 of 553 vs 16 of 545) were observed. Because of the low number of events for these end points, the ProtecT study is not yet definitive for cancer-specific outcomes. The overall number of deaths, primarily from other causes, was identical between the 2 arms, with 55 of 553 and 55 of 545 in the surgery and radiotherapy arms, respectively, and the 95% CI for the hazard ratio (HR) (0.77-1.44) is reasonably narrower. Overall, existing randomized clinical trials provide limited information about the treatment comparison for cancer-specific outcomes, but they provide reasonable evidence that the rates of death from other causes are not too different. In contrast, there have been numerous articles from observational data that provide a range of conclusions for cancer-specific outcomes and for overall survival.^4,5,6,7,8,9,10,11,12,13,14,15,16,17,18,19,20,21,22,23,24,25,26,27,28,29,30^

The biggest threat to the validity of the results from these observational studies is arguably lack of adjustment for confounders. These missing factors may include confounders of oncologic outcomes, such as prostate-specific antigen (PSA) level, Gleason score, perineural invasion (PNI), and cT category, or they may be confounders of death from other causes, such as age and comorbidities. There may also be unmeasured confounders, such as measures of general health and fitness, that alter the choice of treatment and overall survival. Potential advantages of the analysis of observational data (compared with randomized clinical trials) are the larger sample size and the longer follow-up. While larger sample sizes allow better adjustment for confounders and give more precise estimates, larger sample sizes cannot overcome bias if there are unmeasured confounders. In a systematic review and meta-analysis, Wallis et al^4^ demonstrated that the patient characteristics adjusted for and how the adjustment was performed differed across observational studies comparing surgery and radiotherapy for localized prostate cancer. These studies typically adjusted for age, Charlson Comorbidity Index, Gleason score, and cT category in some way, but there was wide variation in the inclusion of other potential confounders, such as PSA level, race, year of diagnosis, and other demographic variables. None of the studies adjusted for prostate gland volume or PNI status.

In addition to the aforementioned issues, existing observational studies vary in their treatment of other key methodological issues and challenges. One issue involves the inclusion of multiple outcomes. Many existing works consider the influence of treatment on overall survival or cause-specific survival.^4,24^ While these outcomes are useful for exploring the aggregate association with treatment, modeling of these outcomes does not give insight into the way in which treatment alters survival. For example, treatment may change the rate of metastasis, or it might alter the rate of death after metastasis. Herein, we consider a unified statistical approach that can incorporate information from multiple outcomes and allow us to identify the association of treatment with different parts of the disease process. In addition, existing works vary in their handling of competing risks, missing data, observation of few events, and differential follow-up between the treatment arms.

The first objective of this article was to explore treatment comparisons at a population level in a retrospective cohort of 4544 patients with prostate cancer with clinically localized disease using statistical methods aimed at addressing these challenges. We used a unified approach based on Bayesian multistate models to analyze the data, which facilitates the consideration of multiple outcomes simultaneously and appropriately handles competing risks and missing data.^30,31,32^ We used a Bayesian estimation approach that, through parameter shrinkage, can incorporate a large number of confounders and address a limited number of events.

Perhaps more important than treatment comparisons at the population level is the identification of specific patients who may benefit more from either surgery or radiotherapy. Even if the treatment has little influence on the overall outcomes in the population, the consequences of treatment on outcomes may likely differ for distinct subgroups of patients. We hypothesized that patient characteristics could be used to generate individualized outcome predictions under each treatment and that these predictions will be useful to identify patients likely to experience superior cancer-specific and/or overall survival outcomes with one treatment over the other.

In the second objective of this article, we developed Bayesian multistate models for individualized treatment predictions. The models can provide predictions for multiple outcomes simultaneously. After validation, this preliminary model can potentially allow for treatment selection to be made based on individualized and treatment-specific probabilities of long-term cancer control and death from other causes. The models can also be used to predict patient outcomes after treatment, which can inform decisions regarding enhanced monitoring after treatment. We developed a web application through which these treatment comparisons can be evaluated.

## Methods

### Patients

All patients with prostate cancer aged 40 to 84 years with clinically localized (including node-negative locally advanced) cT1 to cT3 disease who received either radical prostatectomy or dose-escalated external beam radiotherapy at the University of Michigan between January 1, 1996, and July 1, 2013, were included. Primary analyses were performed in 2017 and 2018. We excluded patients who had Gleason score of 4 or less (n = 14), patients who received chemotherapy before surgery or radiotherapy (n = 15), and patients who initiated any androgen deprivation therapy (ADT) more than 1 year before treatment (n = 21). This left a cohort of 4544 patients. No patients underwent brachytherapy.

The data used for this study are from prospectively collected databases of patients with prostate cancer that are maintained by the departments of urology and radiation oncology at the University of Michigan. This investigation is a retrospective analysis of the databases of patients with prostate cancer. The study followed the Strengthening the Reporting of Observational Studies in Epidemiology (STROBE) reporting guideline. The study received an institutional review board waiver from the University of Michigan and did not require informed patient consent because this is a retrospective study using data from prospectively maintained databases of patients treated at the University of Michigan.

### Outcomes and Baseline Variables

The clinical outcomes were metastatic clinical failure (CF) and death. Both end points are clinically meaningful and may be associated with different factors. Information regarding CF and death was collected prospectively and supplemented by medical record review, and survival data were supplemented by accessing official death records. Clinical failure was defined as the identification of metastatic disease on cross-sectional imaging. Death or survival time was defined as the time from the treatment date (surgery or start of radiotherapy) to the date of death, date of loss to follow-up, 15 years after treatment, or July 1, 2013, whichever was earliest. The time to CF was defined as the time from treatment to the date of CF, date of loss to follow-up, 15 years after treatment, or July 1, 2013, whichever was earliest. Administrative censoring at 15 years was imposed due to less confidence in data quality past 15 years. Additional details on the follow-up are included in the eAppendix in the Supplement.

Covariates included age, prostate gland volume, baseline PSA level, comorbidities, biopsy Gleason score, PNI, cT category, race, and treatment year. Baseline PSA level was the last value for the patient before his first treatment (neoadjuvant hormones [if received], surgery, or radiotherapy). For comorbidities, we used the Charlson Comorbidity Index version that does not include prostate cancer or age as components. All covariates were assessed at baseline and did not vary over time.

We did not adjust for use and duration of adjuvant or pretreatment ADT for patients; therefore, the comparison reflects what was considered appropriate and real-world practice. Use of salvage ADT or radiotherapy was not included in the analysis because this information is unavailable at the time of treatment.

### Statistical Analysis

**Figure 1.**
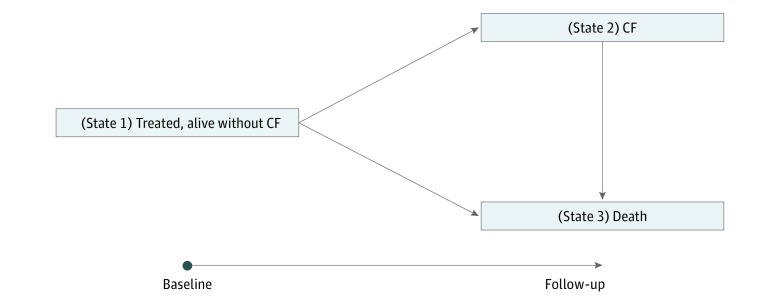
Diagram of Bayesian Multistate Model of Prostate Cancer Bayesian illness-death multistate models showing transitions 1 to 2 (from initial state to metastatic clinical failure [CF]), 2 to 3 (metastatic CF to death), and 1 to 3 (initial state directly to death from other causes).

The progression of patients after initial treatment was described by a Bayesian illness-death multistate model (Figure 1).^30,32^ The model has the following 3 components: the transition from initial treatment to CF, the transition from initial treatment to death from other causes, and the transition from CF to death. Each component was modeled using a Cox proportional hazards regression model, which describes the rate of moving between states and associations with covariates. The multistate model structure allows for the inclusion of multiple potential confounders for cancer-specific outcomes and/or death from other causes, allowing us to reduce or remove bias resulting from treatment allocation and potentially provide a more reliable treatment comparison.

Baseline covariates were included as factors in the 3 transition submodels. Age, log(baseline PSA), and log(gland volume) were centered and/or standardized. Due to the limited number of CF and death events, some variables were grouped or excluded from the submodel for death (D) after CF (transition CF→D). We include the time to CF as a covariate in the model for transition CF→D and use the “clock reset” approach in which time is reset to zero on entering the CF state.^33^ A Cox proportional hazards regression model structure with either a Weibull or piecewise exponential baseline hazard was used for all 3 transitions. The exact form of the models and the covariates are given in the eAppendix in the Supplement. Prior distributions are listed in eTable 1 in the Supplement. In a second analysis used for individual predictions, treatment by covariate interactions was also included as factors in the 3 transition submodels.

Bayesian estimation of the model parameters was performed. Prior distributions were chosen to enforce regularization, which enabled the inclusion of all covariates in the models. Hazard ratios are presented together with 95% CIs. A program using a Markov chain Monte Carlo algorithm was developed in R (R Project for Statistical Computing) to fit this model and provide the posterior distributions for the variables. Variables with missing values were imputed as part of the Markov chain Monte Carlo algorithm using a novel adaptation of the chained equations multiple imputation procedure.^34,35^ This process allowed us to avoid bias arising from removing variables or observations with missingness.^34,35,36^ Additional details, including posterior means for baseline hazard variables, are listed in eTable 3 in the Supplement.

Using the fit of the multistate model that includes interactions, the individualized prediction probabilities (called *state occupancy probabilities*) at any follow-up time *t* can be calculated for any set of baseline covariates. At time *t*, there are 4 possibilities for a potential patient: (1) alive without prior CF, (2) alive with prior CF, (3) died with prior CF, or (4) died without prior CF. The equations for these calculations are presented in the eAppendix in the Supplement. These probabilities are plotted over a range of times from 0 to 15 years and represent the likely outcomes for that patient. The first of the 4 possibilities represents metastasis-free survival. The sum of the first 2 possibilities represents overall survival. We developed an online calculator providing these probabilities (https://lbeesleybiostat.shinyapps.io/ProstatePredictions/). This interactive application presents the probabilities in graphical form, and the user can modify the baseline characteristics to investigate how this modification alters the expected outcomes. The comparison of surgery and radiotherapy at the individual patient level can be based on the probabilities, with the treatment covariate set as either surgery or radiotherapy.

Statistical testing in this study was limited to assessment of HR posterior means and corresponding 95% CIs for all Bayesian multistate model fits. Additional exploration in the eAppendix in the Supplement assesses treatment associations using maximum likelihood estimates and corresponding 95% CIs from standard Cox proportional hazards regression models.

## Results

Of 4544 men (mean [SD] age, 61.2 [8.0] years) in the cohort, 3769 underwent radical prostatectomy, and 775 received external beam radiotherapy. Patients undergoing primary radiotherapy were older, had more comorbidities, and harbored worse or more aggressive tumor characteristics (Table). During the follow-up period, 157 patients (3.5%) had CF, 90 patients (2.0%) died after CF, and 378 patients (8.3%) died of other causes.

**Table.** Descriptive Statistics of Prostate Cancer Data Set Among 4544 Patients

Variable	Surgery	Radiotherapy
No.	3769	775
Age		
Mean (SD), y	59.9 (7.3)	67.6 (8.0)
Missing, No. (%)	0	0
Prostate gland volume		
Mean (SD), mL	39.8 (16.6)	48.4 (27.6)
Missing, No. (%)	1318 (35.0)	256 (33.0)
Baseline PSA level		
Mean (SD), ng/mL	7.5 (8.5)	12.6 (17.3)
Missing, No. (%)	4 (0.1)	0
Charlson Comorbidity Index, No./total No. (%)		
0	2178/3759 (57.7)	421/775 (54.3)
1	330/3759 (8.8)	197/775 (25.4)
2	268/3759 (7.1)	86/775 (11.1)
≥3	77/3759 (2.0)	59/775 (7.6)
Missing	916/3759 (24.4)	12/775 (1.5)
Biopsy Gleason score group, No. (%)		
5/6	1798 (47.7)	240 (31.0)
7 = 3 + 4	1288 (34.2)	225 (29.0)
7 = 4 + 3	394 (10.5)	127 (16.4)
8	164 (4.4)	81 (10.5)
9/10	123 (3.3)	82 (10.6)
Missing	2 (0.1)	20 (2.6)
Perineural invasion, No. (%)	840 (22.3)	268 (34.6)
Missing	30 (0.8)	64 (8.3)
cT category, No. (%)		
1	2679 (71.1)	438 (56.5)
2	1062 (28.2)	292 (37.7)
3	19 (0.5)	41 (5.3)
Missing	9 (0.2)	4 (0.5)
Race, No. (%)		
White	2684 (71.2)	674 (87.0)
African American	170 (4.5)	75 (9.7)
Other	30 (0.8)	6 (0.8)
Missing	885 (23.5)	20 (2.6)
Treatment year, No. (%)		
1996-2000	998 (26.5)	207 (26.7)
2001-2006	1202 (31.9)	376 (48.5)
2007-2013	1569 (41.6)	192 (24.8)
Missing	0	0
Neoadjuvant hormones, No. (%)	211 (5.6)	273 (35.2)
CF (metastasis), No. (%)	102 (2.7)	55 (7.1)
Death, No. (%)	270 (7.2)	198 (25.6)
Death after CF, No. (%)	53 (1.4)	37 (4.8)
Follow-up for death, median (95% CI), y	6.8 (6.5-7.0)	9.1 (8.8-9.8)

Abbreviations: CF, clinical failure; PSA, prostate-specific antigen.

SI conversion factor: To convert PSA level to micrograms per liter, multiply by 1.0.

### Overall Comparison of Surgery vs Radiotherapy

**Figure 2.**
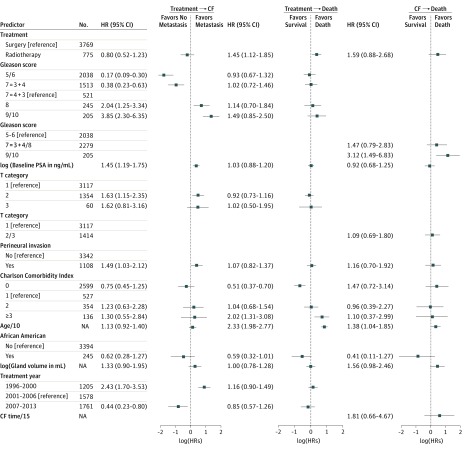
Bayesian Multistate Model Fit to Prostate Cancer Data With Main Effects Only Hazard ratio (HR) estimates from the multistate model for each covariate. For each covariate, the point estimate and 95% CI are shown. CF indicates clinical failure; NA, not applicable; and PSA, prostate-specific antigen. Age/10 and CF time/15 indicate scaling of age and CF time by 10 and 15 years, respectively. To convert PSA level to micrograms per liter, multiply by 1.0.

Figure 2 shows the adjusted HRs for baseline covariates and treatment in each of the 3 transition submodels. The results demonstrate the ability of multistate models to identify what covariates are important for which transitions. The results show some expected patterns. For the transition from initial treatment to CF, cancer-related covariates are associated with CF risk (specifically PSA level, Gleason score, PNI, and cT category). There was no apparent association with age or Charlson Comorbidity Index, but there was a notable association with treatment year, with patients treated more recently having a reduced hazard of CF. After covariate adjustment, there was no significant difference in the hazard of CF for surgery vs radiotherapy (HR, 0.80; 95% CI, 0.52-1.23). In contrast, the unadjusted HR was 2.37 (95% CI, 1.70-3.29).

For the transition directly from initial treatment to death without CF (interpreted as death from other causes), nontumor characteristics (age and higher Charlson Comorbidity Index) were associated with a higher risk of death. Baseline PSA level, Gleason score group, PNI, and cT category were not associated with the risk of death from other causes. Treatment with radiotherapy was associated with increased risk of death from other causes (HR, 1.45; 95% CI, 1.12-1.85). For the transition from CF to death, older age and Gleason score group 9/10 were the only covariates significantly associated with death. eTable 4 in the Supplement compares estimated treatment associations from models with fewer covariates and models with a simpler outcome structure. The unadjusted HR for treatment for death from other causes was 3.60 (95% CI, 2.92-4.43).

### Treatment Comparison for Individual Patients

**Figure 3.**
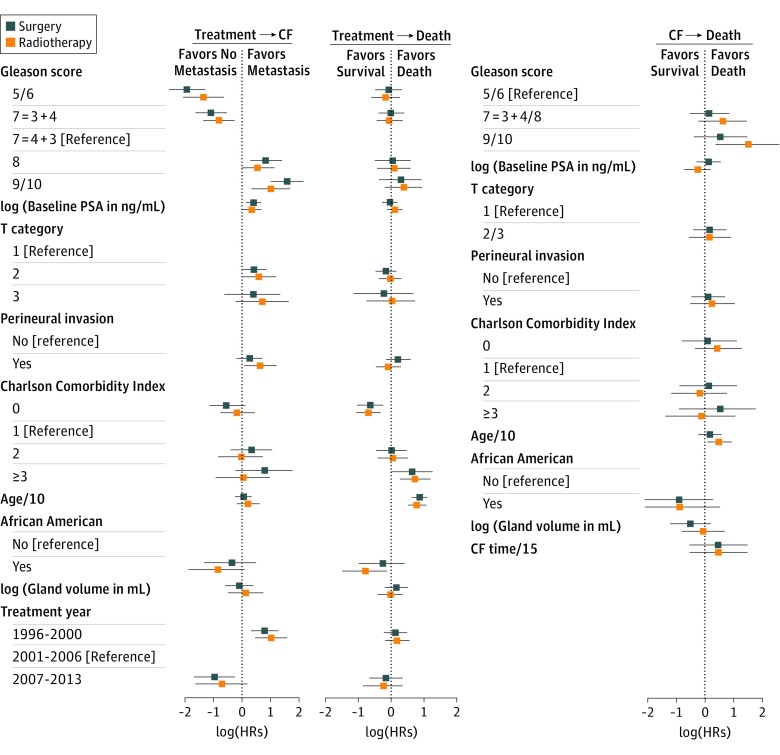
Bayesian Multistate Model Fit to Prostate Cancer Data With Interactions Hazard ratio (HR) estimates from the multistate model for each covariate by treatment. For each covariate, the point estimate and 95% CI are shown. CF indicates clinical failure; PSA, prostate-specific antigen. Age/10 and CF time/15 indicate scaling of age and CF time by 10 and 15 years, respectively. To convert PSA level to micrograms per liter, multiply by 1.0.

The estimated HRs from the model that includes interactions are shown for various variables in Figure 3 and in eTable 2, eFigure 1, and eFigure 2 in the Supplement. Overall conclusions regarding the importance of each covariate in the various transitions did not change. While most interactions were not statistically significant, there was a suggestion of an interaction between treatment and race on the transition from treatment to death and between treatment and Gleason score on the transition to CF.

While covariate-treatment interactions are telling, we were interested in the difference in the predicted outcomes between the 2 treatments for a given patient, which is not immediately clear from the model fit. Using the Bayesian multistate model fit with interactions, we developed a platform for individualized outcome predictions based on treatment, which allows us to visualize and evaluate the influences of treatment on different predicted outcomes over time. The online calculator provides disease state predictions over time given user-specified covariate values.

**Figure 4.**
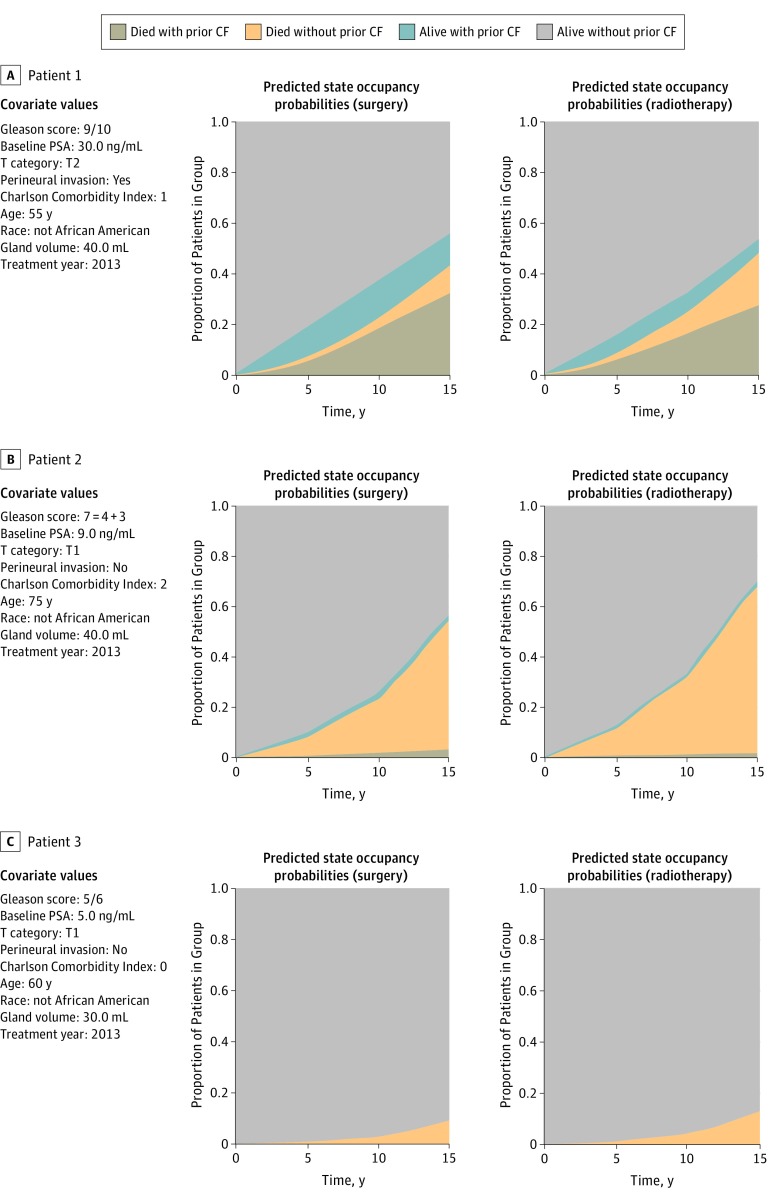
Individual Predictions for Hypothetical Patients Using Bayesian Multistate Models State occupancy probabilities for 3 hypothetical individuals. The first column shows each individual’s characteristics, the second column shows the expected outcome if the individual was to be treated with surgery, and the third column shows the expected outcome if the individual was to be treated with radiotherapy. CF indicates clinical failure; PSA, prostate-specific antigen. To convert PSA level to micrograms per liter, multiply by 1.0.

Figure 4 shows outcome prediction plots generated using the application for 3 hypothetical patients. For each patient, we show the probabilities as if he had been treated with surgery or radiotherapy. The first patient has high-risk disease (younger age, PSA level of 30.0 ng/mL, Gleason score of 9/10, and cT category of T2) (to convert PSA level to micrograms per liter, multiply by 1.0). For him, the likelihood of metastatic recurrence is predicted to be higher with surgery than with radiotherapy, and this patient has a 63% predicted chance of being alive and free of disease at 10 years if given surgery and a chance of 73% if given radiotherapy. The second patient is older (75 years), has more comorbidities (Charlson Comorbidity Index of 2), and does not have advanced disease (PSA level of 9.0 ng/mL, Gleason score of 7 = 4 + 3, and cT category of T1). For this patient, the predicted chance of observing metastasis is low under both treatment arms, but death from other causes is predicted to be higher for radiotherapy. The chance that this patient is alive at 10 years without having experienced recurrence is 75% for surgery and 67% for radiotherapy. The third patient is aged 60 years and has no comorbidities and low-risk disease (PSA level of 5.0 ng/mL, Gleason score of 5/6, and cT category of 1). For this patient, outcomes under both treatments are predicted to be similarly good. The 3 examples were selected to illustrate that there can be modest differences in the predicted outcomes by treatment for some patients.

As an illustration of how the online calculator could be used, we compare state occupancy probabilities supposing that all individuals had received surgery or all individuals had received radiotherapy in eFigures 3, 4, and 5 in the Supplement. The predictions were similar on average, but the relative association of treatment with the predicted outcome for each individual depended on that patient’s characteristics. eFigure 3A in the Supplement shows the 10-year rate of either CF or death among the 2115 patients who had no missing data. If the predicted rates are not small, there is a trend for the probabilities to be lower (better) if treated by surgery. As shown in eFigure 4A in the Supplement, this tendency applies to low-risk and intermediate-risk patients but not high-risk patients. eFigures 3B and 4B in the Supplement show the 10-year rate of CF. When the predicted rates are not small, there is a trend for the CF rates to be lower if treated with radiotherapy than with surgery, particularly for intermediate-risk and high-risk patients. Similar conclusions are shown in eFigure 5 in the Supplement.

## Discussion

Bayesian multistate models allowed us (1) to understand the association between baseline covariates, including treatment, and both metastatic CF and survival in a unified way and (2) to enable individualized predictions of multiple outcomes over time. One of the unique features of multistate models is that they allow different covariates to be important for different transitions. This capability has been illustrated in studies of breast cancer,^37,38,39^ colon cancer,^40,41,42^ and leukemia,^32^ but we are not aware of its being used in prostate cancer before.

Use of this multistate approach provides important insight about the associations between patient and tumor characteristics and key long-term outcomes. In particular, the HRs in Figure 2 and Figure 3 tell a compelling story because more severe tumor characteristics (PSA level, Gleason score, PNI, and cT category) are associated with an increased rate of CF. In contrast, patient characteristics (age and comorbidities) are associated with the hazard of death from other causes. While these are not novel findings, they lend credence to the statistical analysis and may not have been revealed so clearly by other statistical approaches.

Across the cohort as a whole, these data provide no evidence of a difference in risk of metastasis between treatments (HR, 0.80; 95% CI, 0.52-1.23). This finding is consistent with the results from randomized clinical trials.^3^

In contrast, we found a lower rate of death from other causes after surgery compared with radiotherapy. Our result is inconsistent with the findings from the randomized ProtecT study,^3^ and we believe this outcome is due to unmeasured confounders for survival not included in our analyses (at least in part). While we adjusted for age and comorbidities through the Charlson Comorbidity Index, there may be better indexes of health or lifestyle-related factors that are associated with both treatment selection and life expectancy.^42,43^ Differential follow-up and ascertainment of CF and deaths between the treatment groups may also contribute to a differential rate of death from other causes. However, a sensitivity analysis described in the eAppendix in the Supplement did not lead to any substantial change in the findings.

Existing comparisons of surgery and radiotherapy for localized prostate cancer vary in their confounder adjustment and data quality, and our study results tell a generally different story than is seen in many previous observational studies on this topic. Wallis et al^4^ reviewed the findings from 19 studies comparing surgery and radiotherapy for localized prostate cancer in a systematic review and meta-analysis.^5,6,7,8,9,10,11,12,13,14,15,16,17,18,19,20,21,22,23^ They evaluated data quality of the studies in terms of their risk of bias, which was based on the selection of the patients, the comparability between the treatment arms, the assessment of the outcome, and the length of the follow-up. They concluded that most of the studies had a low to moderate risk of bias. However, adjustment for confounding variables was not part of the inclusion criteria for their systematic review, and the adequacy of the adjustment was not a criterion used to evaluate the risk of bias. As discussed in the Introduction section herein, Wallis et al^4^ demonstrated that which patient characteristics were adjusted for and how the adjustment was performed differ across observational studies. The authors found that patients treated with radiotherapy were at an increased risk of overall and prostate cancer–specific mortality compared with patients treated with surgery after adjustment for patient and tumor prognostic factors, but they raised the possibility that their results may have been influenced by residual confounding due to differences between the treatment groups.

Roach et al^24^ explored the influence of data quality and adjustment of confounders on the estimated treatment associations in 14 studies,^2,5,8,11,13,16,19,20,21,25,26,27,28,29^ one of which is a randomized clinical trial. Metrics of data quality explored included data sources (eg, matched single-institution or multi-institution data, nonrandomized observational database, and Surveillance, Epidemiology, and End Results program), sample size, appropriate treatment with ADT, and adjustment for PSA level, comorbidities, Gleason score, and cT category. The quality of those studies varied, and Roach et al^24^ demonstrated that comparisons based on poor-quality data and with minimal adjustment for confounding factors concluded that surgery had superior outcomes. However, analyses based on better-quality data and adjusting for more confounders often demonstrated much smaller differences. In our study, we observed a similar phenomenon in which the estimated treatment associations for both the rate of metastasis and the rate of death from other causes are generally attenuated after adjustment for additional clinical, demographic, and illness-related factors (eTable 4 in the Supplement).

The personalized predictions build on the prevalent framework of nomograms, for which baseline variables are typically prognostic biomarkers. Through the inclusion of interactions in the model, we allowed baseline variables to potentially be predictive biomarkers, meaning they can help inform potential differences in long-term outcomes associated with treatment choice. While most interactions were not statistically significant, there was a suggestion of an interaction between treatment and race, and the widths of the 95% CIs for the interactions were often large in general, so we cannot rule out that some variables could have a large interaction and hence could be predictive of which treatment is better. We also cannot rule out that the unmeasured confounders for the association between treatment and death from other causes could have altered the interactions and hence would also change individualized predictions if they could be adjusted for. However, we believe the online calculator we present is a step toward a more personalized selection of the optimal treatment for each patient, and future validation of this model and its predictive abilities will be needed before direct application to patient-specific treatment decision making.

Another use of the online calculator is to investigate the general characteristics of patients who are most likely to benefit from one treatment or the other. We will investigate this application more fully in future work. However, the results for the model that included interactions (as shown in Figure 3) provide some hints that patients with a high Gleason score (9-10) are likely to benefit more from radiotherapy in terms of metastatic recurrence. In contrast, patients with a low Gleason score (5/6) or PNI may have reduced metastatic recurrence if they receive surgery (eFigures 3, 4, and 5 in the Supplement).

All individuals included in our analysis underwent surgery or radiotherapy as their primary treatment for prostate cancer. During the years of this study, active surveillance was not routinely recommended for low-risk or intermediate-risk men, who thus received the appropriate guideline-concordant care of that era. For some of them, current medical practice would recommend them to active surveillance. For these individuals, a decision between surgery or radiotherapy is less relevant. As a sensitivity analysis, we fit the Bayesian illness-death multistate model with main effects, excluding patients with low-risk prostate cancer. In particular, we defined individuals who would likely receive active surveillance as those with baseline PSA level less than 10 ng/mL, Gleason score of 6, and cT category of 1. This cohort comprised 1399 individuals (30.8% of the total 4544) with low-risk disease. After excluding these patients, we fit the main effects model on the remaining 3145 individuals. The resulting parameter estimates were similar to those presented elsewhere in this article. In particular, the estimated HR of treatment for the rate of CF was 0.73 (95% CI, 0.47-1.14). The estimated HR of treatment for the rate of death from other causes was 1.51 (95% CI, 1.12-2.07), and the HR for death after CF was 1.95 (95% CI, 1.09-3.40). Therefore, we reached similar conclusions regarding the associations between treatment and the various transitions as for the larger model fit that included the low-risk individuals.

### Limitations

There are limitations to our study. Our data came from an observational study and contain unmeasured confounders, loss to follow-up, competing risks, and missing data. These limitations were all addressed to the best of our ability. eTable 4, eFigure 6, and eFigure 7 in the Supplement provide additional explorations into model specification and a sensitivity analysis for potential unmeasured confounding. These limitations notwithstanding, we believe the sophisticated statistical modeling we used has allowed us to reach clinically relevant conclusions that would not have been possible with simpler approaches. We considered a model with treatment-covariate interactions, which influence individual predictions and personalized treatment choices. It is well known that obtaining reliable estimates of interactions requires very large sample sizes. Therefore, our results regarding the interactions are preliminary and would need to be validated with other large studies.

## Conclusions

The online calculator we developed for individualized predictions is the first to date of its type, to our knowledge, that presents multiple outcomes for prostate cancer. With validation, it can serve as a platform to inform treatment selection and posttreatment monitoring in men with localized prostate cancer.

## Notes

Supplement. **eAppendix.** Supplemental Appendix **eTable 1.** Prior Distributions for the Illness-Death Model Parameters **eTable 2.** Estimated Covariate Associations by Treatment in Model With Interactions **eTable 3.** Posterior Means for Baseline Hazard Parameters in Model With Interactions **eTable 4.** Estimated Treatment Hazard Ratio Across Model Specifications **eFigure 1.** Multistate Model Fit With Interactions **eFigure 2.** Estimated Treatment Associations by Patient Characteristics From Model With Interactions **eFigure 3.** Predicted Probability of Clinical Failure/Death by 10 Years (N = 2115, Complete Case Subjects) **eFigure 4.** Predicted Probability of Clinical Failure/Death by 10 Years by Risk Group (N = 2115, Complete Case Subjects) **eFigure 5.** Predicted Probability of Outcome Events by 10 Years by Gleason Score (N = 2115, Complete Case Subjects) **eFigure 6.** Bayesian Illness-Death Model With Fewer Covariates **eFigure 7.** Theoretical and Estimated Associations of Treatment With Other Cause of Death After Accounting for Different Degrees of Unmeasured Confounding **eReferences**
