# Supplementary material for: Individual and Population Comparisons of Surgery and Radiotherapy Outcomes in Prostate Cancer Using Bayesian Multistate Models
Source: JAMA Netw Open. 2019 Feb 1;2(2):e187765. doi: 10.1001/jamanetworkopen.2018.7765 (PMC6484613; doi:10.1001/jamanetworkopen.2018.7765)
Supplement: Supplement. — eAppendix. Supplemental Appendix eTable 1. Prior Distributions for the Illness-Death Model Parameters eTable 2. Estimated Covariate Associations by Treatment in Model With Interactions eTable 3. Posterior Means for Baseline Hazard Parameters in Model With Interactions eTable 4. Estimated Treatment Hazard Ratio Across Model Specifications eFigure 1. Multistate Model Fit With Interactions eFigure 2. Estimated Treatment Associations by Patient Characteristics From Model With Interactions eFigure 3. Predicted Probability of Clinical Failure/Death by 10 Years (N = 2115, Complete Case Subjects) eFigure 4. Predicted Probability of Clinical Failure/Death by 10 Years by Risk Group (N = 2115, Complete Case Subjects) eFigure 5. Predicted Probability of Outcome Events by 10 Years by Gleason Score (N = 2115, Complete Case Subjects) eFigure 6. Bayesian Illness-Death Model With Fewer Covariates eFigure 7. Theoretical and Estimated Associations of Treatment With Other Cause of Death After Accounting for Different Degrees of Unmeasured Confounding eReferences [file jamanetwopen-2-e187765-s001.pdf]

Supplementary Online Content

Beesley LJ, Morgan TM, Spratt DE, et al. Individual and population comparisons of surgery and radiotherapy outcomes in prostate cancer using Bayesian multistate models. *JAMA Netw Open*. 2019;2(2):e187765. doi:10.1001/jamanetworkopen.2018.7765

eAppendix. Supplemental Appendix

eTable 1. Prior Distributions for the Illness-Death Model Parameters

eTable 2. Estimated Covariate Associations by Treatment in Model With Interactions

eTable 3. Posterior Means for Baseline Hazard Parameters in Model With Interactions

eTable 4. Estimated Treatment Hazard Ratio Across Model Specifications

eFigure 1. Multistate Model Fit With Interactions

eFigure 2. Estimated Treatment Associations by Patient Characteristics From Model With Interactions

eFigure 3. Predicted Probability of Clinical Failure/Death by 10 Years (N=2115, Complete Case Subjects)

eFigure 4. Predicted Probability of Clinical Failure/Death by 10 Years by Risk Group (N=2115, Complete Case Subjects)

eFigure 5. Predicted Probability of Outcome Events by 10 Years by Gleason Score (N=2115, Complete Case Subjects)

eFigure 6. Bayesian Illness-Death Model With Fewer Covariates

eFigure 7. Theoretical and Estimated Associations of Treatment With Other Cause of Death After Accounting for Different Degrees of Unmeasured Confounding

eReferences

This supplementary material has been provided by the authors to give readers additional information about their work.

eAppendix. Supplemental Appendix

Variable Definitions

The Charlson index for comorbidities is based on 22 conditions. Each condition is assigned a score of 1, 2, 3, or 6, depending on the risk of dying associated with each one. Scores are summed to provide a total score.

1 point each: Myocardial infarct, congestive heart failure, peripheral vascular disease, dementia, cerebrovascular disease, chronic lung disease, connective tissue disease, ulcer, chronic liver disease, diabetes.

2 points each: Hemiplegia, moderate or severe kidney disease, diabetes with end organ damage, tumor, leukemia, lymphoma.

3 points each: Moderate or severe liver disease.

6 points each: Malignant tumor, metastasis, AIDS.

The prostate cancer malignancy was not included as a score in this index.

The Gleason score used in this study is from a biopsy prior to surgery or radiation. By using the biopsy result for both surgery and radiation patients, we ensure comparable variables. PSA is measured from a blood draw prior to surgery and radiation and also prior to initiation of any hormone therapy. To reduce the possibility of residual confounding, we did not apply cut-points or thresholds to continuous variables in the statistical model.

© 2019 Beesley LJ et al. *JAMA Network Open.*

Patient Follow-up

We defined the censoring date as the minimum of 15 years post-baseline, July 1 2013, and the date of loss to follow-up. We define the date of last follow-up as the last time in which there was some contact with the patient (for example, via phone call) or some contact with the patient's home doctor. This can be viewed as the date of last contact. In particular, patients were followed at The University of Michigan with approximately annual visits. Imaging was used to determine metastatic clinical failure and was called for when PSA values or other clinical signs suggested possible metastases. For patients not being followed at the University of Michigan, the patients and their physicians were contacted to ascertain current disease status. National and State death indexes were queried to ascertain the alive/dead status of patients who were lost to follow up.

A sensitivity analysis (described in a section below) was performed to explore the impact of choices regarding censoring of metastasis and death on inference.

© 2019 Beesley LJ et al. *JAMA Network Open.*

Details of the Multistate Model and Estimation Method

Figure 1 in the main paper shows the structure of the multistate model used in the main paper. All of the transitions in the multistate model assume a proportional hazards regression model. For transitions 1 to 2 (initial state to CF) and 2 to 3 (CF to death), we assume Weibull baseline hazard rates with cumulative baseline hazards of the form $H_0(t) = (t/\lambda)^\rho$. For transition 1 to 3 (initial state directly to death), we assume a piecewise exponential baseline hazard with cut-points at 5, 10 and 12.5 years with values of the hazard between the cut-points of b1, b2, b3 and b4.

For fitting the model, some of the variables are centered, scaled or grouped. Age is centered at 60 and scaled by 10 years. Log(PSA) is centered at 1.8. log(Gland Volume) is centered at 3.6. All logarithms are to base e. For the 2 to 3 transition model, clinical failure time is a covariate and it is scaled by 15 years, the treatment year group was not included, T stages 2 and 3 were combined and Gleason grades 3+4, 4+3 and 8 were combined. The variable groupings for this transition was chosen in order to obtain good model fit and good numerical properties of the parameter estimation.

We consider two formulations of this model. The first formulation includes only main effects, and the resulting model fit is discussed in the main paper. The second formulation of the model also includes interactions with treatment for each predictor in each one of the multistate model transitions. For the model with interactions, continuous versions of the categorical ordered variables are used for the interaction terms. This applies to Gleason, stage and comorbidities (i.e. for Gleason it is a continuous variable taking the values 0,1,2,3 and 4, rather than being represented as 4 dummy variables).

There are many regression coefficients to estimate, and for this situation some form of shrinkage or regularization is beneficial to give less variable parameter estimates. The shrinkage is achieved through the choice of the variance of the prior distributions. The prior distributions are chosen to impose a very mild amount of shrinkage on the main effect parameters. For the situation of the CF to death transition where there are fewer subjects and thus less information in the data, a modest amount of shrinkage is applied. For the model with interactions, a stronger amount of shrinkage is applied to the interaction terms because a priori it is unlikely that any of the treatment-by-covariate interactions will be large. We note that varying the prior variances within a reasonable range did not change any of the conclusions of the analyses. **eTable 1** shows the prior distributions chosen for each model parameter.

A custom-built program was developed in R to fit both formulations of the multistate model. In this program, the likelihood contribution from each subject was combined with the prior information, and a Markov Chain Monte Carlo (MCMC) algorithm was run for 20000 iterations with a burn-in of 3000 iterations to provide draws from the posterior distributions for all the parameters. The goodness-of-fit of the model was assessed by comparing expected and model predicted survival curves and by examining Cox-Snell residuals. These diagnostics indicated good model fit.

Within each iteration of the MCMC estimation algorithm, we performed imputation of the missing covariate values. For the imputation procedure for the missing baseline covariates, we used the following sequential procedure. For each covariate with missing values, the missing value for a given patient is drawn from its conditional posterior predictive distribution. This conditional distribution is derived from a product of the likelihood of the outcome (CF and death) data and a model for the conditional distribution of that covariate given the other baseline covariates. Regression models (either logistic, cumulative logistic,

© 2019 Beesley LJ et al. *JAMA Network Open.*

multinomial, or linear) were specified for each baseline variable with missing values, conditional on the other baseline variables. This imputation strategy was developed in Bartlett et al. (2014) and has a strong theoretical motivation.[1] The baseline covariate models were cumulative logistic models for Gleason, T Stage, and Charlson comorbidities. The baseline covariate models were linear models for log(PSA) and log(Gland Volume), and they were logistic models for PNI and race (African-American vs. White/Other). Additional information about this imputation strategy can be found in Bartlett et al. (2014) and Beesley et al. (2016).[1,2]

Main Effects Model

We present the results of fitting the main effects only version of the multistate model to the prostate cancer data in the main paper (along with a model including interactions with treatment). We discuss the main effects model fit in the main paper, but we include some additional comments here.

The hazard ratios in **Figure 2** in the main paper tell a compelling story, as tumor characteristics (clinical T-stage, Gleason, PSA, PNI) are associated with the transition to clinical failure, with a clear monotonically increasing risk associated with increasing Gleason grade. In contrast, patient characteristics (age, comorbidities) are associated with the hazard of death from other causes, with a clear monotonically increasing risk with increasing Charlson score. While these are not novel findings, they lend credence to the statistical modeling.

We note in the main paper that we obtain a lower rate of death from other causes in the surgery group compared to the radiation group. Because this is inconsistent with overall survival data from the randomized ProtecT study, we believe our finding is likely due to other unmeasured confounders not included in the analyses. While we did adjust for age and comorbidities (Charlson Index), inclusion of additional health or lifestyle-related factors may impact the estimated rates of death from other causes in each of the treatment groups.

Another possible factor that might contribute to the explanation of a different hazard of death from other causes between surgery and radiation is differential follow-up and differential ascertainment of CF and deaths in our data. In our study, surgery patients tended to travel a greater distance for their treatment and often received longitudinal follow-up in their own community, whereas radiation patients more often continued their long-term follow-up at the University of Michigan. We defined the censoring date as the minimum of 15 years post-baseline, July 1 2013, and the date of loss to follow-up. In our data, the surgery patients did have a higher rate of drop-out from active follow up, so we defined the date of loss to follow-up to include follow-up outside of active visits to the University of Michigan clinics. We considered two general methods for defining the follow-up time. In Method 1, we define censoring for clinical failure as the date at which we are very confident at which prior metastasis did not occur based on active follow-up. This was defined using UM medical charts and other sources. In Method 2, we define the date of last follow-up as the last time in which there was some contact with the patient (via phone call or through a visit to a different UM clinic) or some contact with the patient's home doctor. This can be viewed as the date of last contact. We use Method 2 to define loss to follow-up for our primary analysis as this allows censoring to be more equivalently defined for surgery and radiation patients. We performed sensitivity analyses where we modified this assumption and used Method 1 to define earlier censoring dates for CF. Repeat analyses were performed with overall survival time redefined as the time from the treatment date to the date of death or last date of contact (censoring Method 2) and time to clinical failure

© 2019 Beesley LJ et al. *JAMA Network Open.*

redefined as the time from treatment date to the date of clinical failure or the last date the patient was seen in a clinical setting without clinical failure (censoring Method 1). We obtain similar results. This modified analysis did not lead to any substantial change in the findings.

Unlike some previous observational studies, we did not find a significant association between treatment and the rate of metastasis in our multistate modeling. We attribute this to our ability to adjust for a variety of patient and tumor characteristics. We note that if we crudely compare the Kaplan-Meier estimator of time to clinical failure between the two treatment groups, we do see a difference in the metastasis rate between treatments (**eTable 4**). This significant association disappears when we adjust for these additional patient and tumor characteristics.

It is interesting that the time from initial treatment to CF is not associated with the time from CF to death. We might have expected that those who had a short time to CF would also have had a short time from CF to death, because they have inherently aggressive disease. However, we did not find such an association. It is possible that after adjusting for factors such as Gleason, T stage and PSA there is no remaining association. Generally, we found that tumor and patient characteristics were less important for the transition from CF to death compared to the other two transitions. Compared to the whole dataset, there were a limited number of patients who make this transition, which limits the power to detect any strong associations. Furthermore, the treatments the patients may receive after CF can vary widely, and the effect of these treatments would likely be more important than baseline covariates, which were collected possibly many years previously.

The lower hazard of clinical failure for patients treated more recently compared to patients treated at earlier times is notable. We believe this can be attributed to drifts in time in the Gleason grade, the clinical T-stage, imaging techniques, the surgical and radiation therapy techniques and changes over time in the use of adjuvant or salvage therapies.

Model with Interactions

We fit the proposed illness-death model to the prostate cancer data using the model with interactions. **eFigure 1** and **eTable 2** show the mean covariate associations and corresponding 95% credible intervals. A reformulation of these parameters showing the covariate associations by treatment can be found in the main paper. This plot shows that the covariate associations tend to be similar between the radiation and the surgery groups. The only variables where there is a suggestion of a differential association are Charlson, PNI, and Gleason for the transition to CF and race for the transition to death from other causes.

We note that the baseline event rates for the 1->2 and 2->3 transitions are assumed to be Weibull, and the baseline event rates for the 1->3 transition is assumed to be piecewise exponential, which translates into a piecewise constant baseline hazard for this transition. **eTable 3** shows the posterior means for the baseline hazard parameters. The posterior mean for the main effect of treatment is 0.384 (95% CI: -0.466, 1.190) for the 1->2 Transition, 0.414 (95% CI: -0.069, 0.866) for the 1->3 Transition, and 0.058 (95% CI: -1.048, 1.195) for the 2->3 Transition.

© 2019 Beesley LJ et al. *JAMA Network Open.*

State Occupancy Probabilities

After we fit the model, we may be interested in using the model estimates to predict outcomes for future patients given covariates. For example, we may want to estimate the probability that an individual has died and/or has experienced a clinical failure by various times after baseline using the patient's baseline information. These probabilities may be useful for predicting patient prognosis or exploring the potential relationship between treatment decisions and prognosis. Below, we present equations for estimating several probabilities of interest.

These probabilities are depicted in the online calculator. State occupancy probabilities represent the expected fraction of patients (with specified characteristics) who will be in the each of the four possible states at any follow-up time t. The four possible states are (1) had a CF and then died, (2) had a CF but still alive, (3) alive without CF, and (4) died without prior CF. Using the multistate illness-death model, we can derive equations for estimating the state occupancy probabilities for a given set of patient characteristics and treatment assignment. These equations will depend on the parameter estimates from the multistate model. The means of the posterior distributions from the model that includes interactions are used as the parameter estimates in the online calculator. The posterior distributions of the parameters are obtained by fitting the multistate illness-death model to the data for 4544 patients.

Here, we present the equations used to estimate the state occupancy probabilities given baseline characteristics and a treatment assignment. First, we clarify some notation. Let X be the set of patient characteristics and the treatment assignment. Let X1, X2, and X3 be the subsets of X used in the models for the transitions from state 1 to state 2, state 1 to state 3, and state 2 to state 3 respectively. The three transitions in the illness-death model have hazards as follows. The hazard of clinical failure at time t (from states 1 to 2) can be written as $\lambda_{12}(t) = \lambda_0(t)e^{\beta_{12}X_1}$ where $\lambda_0(t)$ denotes the baseline hazard at time t. The hazard of death at time t without prior clinical failure (from states 1 to 3) can be written as $\lambda_{13}(t) = \lambda_0(t)e^{\beta_{13}X_2}$. The hazard of death at time t after prior clinical failure at time f (from states 2 to 3) can be written as $\lambda_{13}(t-f|f) = \lambda_0(t-f)e^{\beta_{23}X_3+\beta_f f}$ where β_f is the parameter in the transition from states 2 to 3 corresponding to time of clinical failure. Denote the cumulative hazards by $\Lambda_{jk}(t) = \int \lambda_{jk}(u)du$.
The state occupancy probabilities are given by

$$P(\text{ dead before time } t \text{ with prior CF } |X) = \int_0^t \left[1 - e^{-\Lambda_{23}(t-u|u)}\right] \lambda_{12}(u)e^{-\Lambda_{13}(u)-\Lambda_{12}(u)}\, du$$

$$P(\text{ alive at time } t \text{ with prior CF } |X) = \int_0^t e^{-\Lambda_{23}(t-u|u)}\lambda_{12}(u)e^{-\Lambda_{13}(u)-\Lambda_{12}(u)}\, du$$

$$P(\text{ alive at time } t \text{ with no history of CF } |X) = e^{-\Lambda_{13}(t)-\Lambda_{12}(t)}$$

$$P(\text{ dead before time } t \text{ with no history of CF } |X) = \int_0^t \lambda_{13}(u)e^{-\Lambda_{12}(u)-\Lambda_{13}(u)}\, du$$

Using the probabilities above, we have that

$$P(\text{ dead before time } t\,|X) = P(\text{ dead before time } t \text{ with prior CF } |X) + P(\text{ dead before time } t \text{ with no history of CF } |X)$$

© 2019 Beesley LJ et al. *JAMA Network Open.*

Additional Exploration of Multistate Model with Interactions

In this section, we explore some implications of the multistate model fit with interactions in terms of treatment comparisons within covariate subgroups. We have a significant interaction between Gleason score and treatment for the Treatment->CF transition, and we have some hints of other interactions. **eFigure 2** shows the model-estimated estimated treatment associations for some covariates of interest. It is important to note that, although there are some trends suggesting some treatment interactions might be important, a larger study is needed to definitively identify which covariates are predictive of treatment outcome. While we may see covariate-treatment interactions, it is not clear the aggregate impact these associations will have on predicted outcomes. In this section, we briefly explore model predictions for subjects with different characteristics. For this exploration, we consider the complete case subset of the patients (N=2115). For each subject, we calculate his probability of having different outcome events by 10 years given his individual characteristics and supposing he had received either radiation or surgery. On average, there is not much difference between the predicted outcomes by treatment.

While we do not see a large overall difference in the event rates between the two treatment groups, we may expect there to be appreciable treatment associations on the predictions for some individual subjects or groups of subjects. **eFigure 3** shows the predicted probability of (1) clinical failure or death by 10 years and (2) observed clinical failure by 10 years for each of the N=2115 complete case subjects. The predictions look different for each individual, and the relative merit of surgery or radiation will vary from individual to individual. When we look only at the clinical failure outcome (whether subjects are alive with clinical failure or died with clinical failure) in **eFigure 3b**, many subjects appear to benefit from radiation over surgery. Differences can also be seen in the comparative (by treatment) predicted rates of any event (which includes death from other causes) across subjects, as shown in **eFigure 3a**. As mentioned previously, our model predicts that the rate of death from other causes is higher in the radiation group compared to the surgery group (although it is not significant in the model with interactions). Due to results from the ProTect trial, we do not expect the rates of death from other causes to differ much in reality, and we believe our result is due to residual unmeasured confounding. In future work, we will explore this issue further in order to obtain patient-specific predictions that are more representative of the potential outcomes for that given patient.

Suppose we separate out subjects by their risk based on baseline variables. Here, we define subjects to be high risk if they have T3 disease, Gleason 8-10, or PSA over 20 and low risk if they have T1 disease and a Gleason <= 6 and PSA less than 10. Subjects with higher risk categories exhibit markedly worse prognosis. We compare the probabilities of (1) prior clinical failure or death and (2) prior clinical failure only at 10 years by risk status in **eFigure S4**. As shown in **eFigure 4a**, when predicted clinical failure or death rates are small, there is little difference between the treatments. However, for larger predicted rates, there is a trend for the probabilities to be lower (better) if treated by surgery, particularly for low and moderate risk patients. In contrast, **eFigure 4b** shows that there is a trend for the observed clinical failure rates to be lower if treated with radiation than with surgery, particularly for moderate and high risk patients.

If we compare these probabilities by Gleason score (**eFigure 5**), we can see a trend where subjects with higher Gleason score are more often predicted to benefit from radiation over surgery. We see this trend whether we consider just clinical failure outcomes or any

© 2019 Beesley LJ et al. *JAMA Network Open.*

event. Overall, our model predicts the majority of subjects will benefit from surgery over radiation for any event and that many subjects will benefit from radiation for the clinical failure outcome specifically, and this is due to a differential rate of death from other causes by treatment in our estimated model.

© 2019 Beesley LJ et al. *JAMA Network Open.*

Sensitivity Analysis Excluding Subjects Likely to Receive Active Surveillance

All subjects included in our analysis received either surgery or radiation therapy as their primary treatment for prostate cancer. For many of these subjects, current medical practice would assign some of these subjects to active surveillance. For these subjects, a decision between surgery or radiation is less relevant. As a sensitivity analysis, we fit the Bayesian illness death model with main effects only excluding subjects with very low risk prostate cancer. In particular, we define subjects who would likely receive active surveillance as those subjects with Gleason score 6, baseline PSA less than 10, and with clinical T1. This consists of 1399 subjects (30.7% of the total 4544) with very low risk disease. After excluding these subjects, we fit the main effects model on the remaining 3145 subjects. The resulting parameter estimates are extremely similar to those presented in this paper. In particular, the estimated hazard ratio of treatment for the rate of clinical failure is 0.73 (95% credible interval 0.47, 1.14). The estimated hazard ratio for treatment for the rate of death from other causes is 1.51 (1.12, 2.07), and the hazard ratio for death after clinical failure is 1.95 (1.09, 3.40). We therefore reach similar conclusions regarding the associations between treatment and the various transitions as for the larger model fit including the low risk subjects.

© 2019 Beesley LJ et al. *JAMA Network Open.*

Sensitivity Analysis Excluding Subjects with Low and Very High Surgery Propensities

In order for the proposed treatment decision-making tool to be useful, subjects must be eligible to receive either treatment. In this analysis, we wanted to restrict our attention to patients who could conceivably receive either treatment. We noted previously that the subjects in our sample receiving radiation tended to have worse Charlson scores and Gleason scores among other things. In order to fairly compare the treatments amongst patients who could conceivably receive either treatment, we restricted our analyses to subjects age 40-84 and cT1-3. For the resulting sample, we compared the covariate distributions between the treatment groups, and there was a lot of overlap. Additionally, a logistic regression for P(surgery given covariates) again indicated reasonable overlap in the range of estimated probabilities between subjects who received surgery and who received radiation. We similarly restricted the entries in our online calculator to subjects with covariate values consistent with our analytical sample. These choices allow us to focus our attention on subjects who could conceivably receive either treatment, which is our population of interest in this study.

Even after the above restrictions, there was a small remaining subset of patients who would not be considered candidates for surgery based on tumor or clinical characteristics. A comparison of treatment outcomes is of minimal interest for this group of patients. As a sensitivity analysis, we evaluated the treatment associations excluding all subjects who have a low (<0.25) or very high (>0.98) propensity for receiving surgery. There were 74 patients (5 surgery patients and 69 radiation) for whom the probability of surgery was less than 0.25 (based on a logistic regression model fit to the imputed data). There were 379 patients (370 surgery patients and 9 radiation) for whom the probability of surgery was greater than or equal to 0.98.

We fit the Bayesian multistate model to the remaining 4091 patients, which resulted in a hazard ratio estimate for treatment for the initial state to clinical failure of 0.82 (95% CI = (0.53, 1.28)) and a hazard ratio for treatment from the initial state to death from other causes transition of 1.40 (95% CI = (1.07,1.89)). These results are similar to the results from the full dataset, and the overall conclusions are not altered.

© 2019 Beesley LJ et al. *JAMA Network Open.*

Comparison with Simpler Model Fits

In this section, we compare our multistate model fit to modeling using (1) model for overall survival or (2) a model with fewer or no covariates (besides treatment). We consider model fits without interactions with treatment.

eTable 4 compares the estimated treatment associations for several model fits including the covariate set included in the main effects illness-death model explored previously, an illness-death model with fewer or no covariates, and several Cox regression model fits for Overall survival and for the individual transitions. For simplicity and ease of computation, we use complete case analysis for the simple (non-Bayesian) Cox model fits. The simpler set of covariates are treatment, age, Charlson (Yes/No) and risk (Low, Moderate, High). Low risk is defined as Gleason<= 6 and PSA<10 and cTstage=1, high risk is defined as Gleason 8-10 or PSA>20 or cTstage=3. With fewer covariates in the illness-death model, the estimated effect of treatment for the transition to death from other causes (1->3) increases. This estimated hazard ratio further increases when no covariates are included in the model, indicating that incorporation of additional covariates resulted in a weaker estimated association of treatment for this transition. The same is true for the transition to death after clinical failure (2- > 3). For the treatment association for the transition to clinical failure (1->2), we see no significant treatment association under both adjusted illness-death models. However, a model that only includes treatment as a predictor would suggest that subjects receiving radiation have significantly higher rates of clinical failure compared to subjects receiving surgery. We note that in general, the intervals obtained using the Bayesian estimation tend to be narrower compared to the estimation based on the Cox model fit to complete case data. One reason for this is that the Bayesian model fits use all of the available data in the estimation. Another reason is that the Bayesian models allow us to borrow information across transitions to help with imputation and provide a way to impose shrinkage for parameters with small expected associations (e.g. in the transition to death after clinical failure).

Many existing comparisons of surgery and radiation for treatment of localized prostate cancer in the literature consider overall survival as the primary outcome. In our data, we see that modeling of overall survival results in a significant treatment association, even after covariate adjustment. In contrast, our main illness-death model explored in this paper indicates that there is a significant association of treatment with the rate of death from other causes, but we see no significant association of treatment with the rate of clinical failure. A model of overall survival involves a combination of disease-related and non-disease-related associations, and it therefore gives a less clear picture of the association between treatment and survival. This provides some motivation for use of an illness-death model rather than an aggregate model for overall survival.

We note that the two adjusted Bayesian illness-death model fits seen in **eTable 4** come to generally similar conclusions regarding the treatment associations with the various transitions. However, we do see some differences in the point estimates, particularly for the 1->2 transition. While the estimated treatment associations tell a broadly similar story, the associations of the other model covariates for the illness-death model with fewer covariates suggest some motivation for additional covariate adjustment. **eFigure 6** shows the estimated credible intervals for illness-death model parameters under the smaller set of covariates. We note that the association of Charlson score on the transition to clinical failure is significant, indicating that subjects with nonzero Charlson score tend to have

© 2019 Beesley LJ et al. *JAMA Network Open.*

higher rates of clinical failure. In contrast, results from the illness-death model with the larger covariate set do not indicate any association of Charlson score with that transition, and we might a priori believe that there should not be such an association. Additionally, the model with the larger covariate set identifies some covariates with very strong effects on the transition to clinical failure that are not included in the model with fewer covariates (e.g. perineural invasion and treatment year). Finally, while additional covariates may not dramatically change the inference for treatment associations, the inclusion of additional covariates may have a substantial impact on personalized predictions, where additional covariates such as Gleason score may provide a more refined stratification of patient risk. Therefore, there is some evidence and intuition that a model with these additional covariates may better capture the associations present in our data.

© 2019 Beesley LJ et al. *JAMA Network Open.*

Exploration of Unmeasured Confounding

In this section, we perform some sensitivity analyses to investigate whether the association between treatment and death from other causes could be explained by an unmeasured confounder. By “confounder”, we are referring to an unmeasured variable U that is associated with both the treatment selection and the rate of death from other causes, adjusting for all the other predicted included in the model for death from other causes.

We may believe that we have captured many major factors related to death from other causes, such as age and comorbidities (Charlson score). There may be other socioeconomic or comorbidity-related factors that we did not adjust for, but we may believe that the corresponding odds ratio for the association with treatment and the hazard ratio for the association with the outcome may be small to moderate (for example, between 0.60 and 1.64).

We explore the treatment association we might estimate if we had accounted for an unmeasured confounder U related to both the other cause death rate and treatment selection using a small study in which this new variable U is an additional covariate to be included in the analysis. For simplicity, we assume U is independent of all predictors except treatment assignment. We focus on our complete case subjects.

We generate 9 datasets as follows: For each subject, we generate $U \sim N(\alpha, 1)$ for subjects receiving radiation and $U \sim N(0,1)$ for subjects receiving surgery. One can show that α represents the log-odds ratio association between U and treatment assignment given other covariates assuming that U is independent of other covariates. Here, values of α further away from zero indicate stronger association. For each dataset, we fit a Bayesian illness-death model with offset term γU included in the model for death from other causes. This imposes association γ between U and the rate of death from other causes. We then estimate the treatment association and credible or confidence intervals. We perform this procedure for different combinations of $\alpha = (0.2, 0.5, 0.8)$ and $\gamma = (0.2, 0.5, 0.8)$. We focus on positive values for both parameters, and we obtain similar results when considering associations in the opposite direction. Results are shown in **eFigure 7.**

The setting in which both α and γ are zero corresponds to the setting with no unmeasured confounding. When both are nonzero, however, the resulting estimate for the treatment association on the rate of death from other causes is impacted. For positive values of α and γ, this results in a shift toward and beyond zero. These results suggest that it is plausible that we would have observed a null association if we had accounted for unmeasured U in certain settings.

In particular, these results suggest that if one of the associations (α or γ) is at least 0.2 and the other is 0.5 then the *95% credible interval* includes zero. Values of 0.2 and 0.5 correspond to an odds ratio or hazard ratio between 1.22 and 1.64, which would be viewed as a modest degree of association. In order for the *point estimate* to move to exactly zero, we require stronger associations between U and the other cause death rate and between U and treatment assignment. A point estimate of zero is achieved if one of the associations (α or γ) is 0.5 and the other is 0.8. The degree to which we believe such unmeasured confounding may exist may be a topic for further discussion. For comparison, age/10 has a log-HR of about 0.85 for the same transition, and the treatment association with age/10 is represented by a log-OR of -1.25 for α (note: we can ignore the direction and focus on

© 2019 Beesley LJ et al. *JAMA Network Open.*

the magnitude). Overall, we require reasonably strong associations for U (but weaker than observed associations for age/10) in order to *totally* explain away the estimated association for the association between treatment and death from other causes. This suggests that, while unmeasured confounding may certainly explain the estimated association between treatment and other causes death in part, it may not necessarily completely explain away this association.

In addition to presenting estimated treatment associations in **eFigure 7**, we also compare these estimates to some theoretical bounds presented in Ding and VanderWeele (2016) in *Epidemiology* and VanderWeele and Ding (2017) in *Annals of Internal Medicine*.[3,4] In these works, the authors derive the weakest estimated association we can get due to the unmeasured confounder based on different levels of association between the unmeasured confounder and the treatment assignment and outcome. As demonstrated by **eFigure 7,** our simulation-based results agree with the theoretical bounds.

Tables and Figures

eTable 1. Prior Distributions for the Illness-Death Model Parameters

Parameter	Prior Distribution
$\log(\lambda)$ for the 1 to 2 Transition	$N(0, 4^2)$
$\log(\lambda)$ for the 2 to 3 Transition	$N(0, 4^2)$
ρ for the 1 to 2 Transition	$\Gamma(2.5, 0.4)$
ρ for the 2 to 3 Transition	$\Gamma(2.5, 0.4)$
$b^t s$ for the 1 to 3 Transition	$\Gamma(0.0625, 1.6)$
Main Effects for the 1 to 2 Transition	$N(0, 2^2)$
Main Effects for the 1 to 3 Transition	$N(0, 2^2)$
Main Effects for the 2 to 3 Transition	$N(0, 1.5^2)$
Interactions for the 1 to 2 Transition	$N(0, 0.5^2)$
Interactions for the 1 to 3 Transition	$N(0, 0.5^2)$
Interactions for the 2 to 3 Transition	$N(0, 0.5^2)$

© 2019 Beesley LJ et al. *JAMA Network Open.*

eTable 2. Estimated Covariate Associations by Treatment in Model With Interactions

	Transition 1->2 HR (95% Credible Interval)		Transition 1->3 HR (95% Credible Interval)	
	**Surgery**	**Radiation**	**Surgery**	**Radiation**
T Stage				
T1 (ref)				
T2	1.51 (1.02, 2.25)	1.78 (1.01, 3.16)	0.85 (0.63, 1.13)	0.96 (0.69, 1.32)
T3	1.47 (0.55, 3.64)	2.04 (0.81, 4.90)	0.79 (0.32, 1.90)	1.03 (0.48, 2.02)
Charlson Score				
0	0.57 (0.33, 1.08)	0.83 (0.48, 1.49)	0.52 (0.36, 0.75)	0.50 (0.35, 0.70)
1 (ref)				
2	1.38 (0.68, 2.75)	0.95 (0.44, 1.99)	1.01 (0.64, 1.56)	1.06 (0.68, 1.62)
3+	2.20 (0.82, 5.53)	1.04 (0.40, 2.52)	1.89 (1.02, 3.42)	2.07 (1.31, 3.23)
Gleason Score				
5-6	0.14 (0.08, 0.26)	0.26 (0.13, 0.51)	0.92 (0.63, 1.34)	0.84 (0.56, 1.27)
7=3+4	0.33 (0.20, 0.56)	0.44 (0.26, 0.74)	1.00 (0.70, 1.45)	0.95 (0.66, 1.38)
7=4+3 (ref)				
8	2.27 (1.38, 3.84)	1.70 (1.00, 2.97)	1.05 (0.61, 1.74)	1.10 (0.67, 1.78)
9-10	4.81 (2.76, 8.40)	2.71 (1.41, 5.12)	1.35 (0.71, 2.45)	1.48 (0.83, 2.52)
Perineural Invasion				
No (ref)				
Yes	1.29 (0.84, 1.95)	1.86 (1.08, 3.23)	1.23 (0.86, 1.75)	0.91 (0.63, 1.31)
Age/10	1.05 (0.80, 1.37)	1.22 (0.85, 1.77)	2.37 (1.92, 2.94)	2.19 (1.69, 2.82)
Log(Baseline PSA in ng/mL)	1.48 (1.17, 1.87)	1.40 (1.03, 1.90)	0.95 (0.77, 1.16)	1.11 (0.90, 1.38)
Treatment Year				
1996-2000	2.17 (1.41, 3.41)	2.73 (1.60, 4.65)	1.14 (0.82, 1.57)	1.19 (0.85, 1.68)
2001-2006 (ref)				
2007-2015	0.38 (0.19, 0.76)	0.49 (0.20, 1.16)	0.85 (0.52, 1.37)	0.80 (0.43, 1.41)
African American				
No				
Yes (ref)	0.69 (0.27, 1.55)	0.43 (0.15, 1.04)	0.78 (0.37, 1.48)	0.45 (0.22, 0.85)
Log(Gland volume in cc)	0.90 (0.56, 1.41)	1.13 (0.62, 2.04)	1.17 (0.84, 1.62)	0.98 (0.68, 1.40)

	Transition 2->3 HR (95% Credible Interval)	
	**Surgery**	**Radiation**
T Stage		
T1 (ref)		
T2/3	1.18 (0.68, 2.05)	1.16 (0.58, 2.47)
Charlson Score		
0	1.10 (0.46, 2.94)	1.53 (0.72, 3.51)
1 (ref)		
2	1.15 (0.41, 2.96)	0.83 (0.31, 2.08)
3+	1.71 (0.41, 5.69)	0.88 (0.25, 0.51)
Gleason Score		
5/6/7=3+4 (ref)		
7=4+3/8	1.14 (0.60, 2.28)	1.87 (0.80, 4.24)
9/10	1.72 (0.69, 4.26)	4.60 (1.49, 13.2)
Perineural Invasion		
No (ref)		
Yes	1.11 (0.64, 1.95)	1.29 (0.61, 2.77)
Age/10	1.19 (0.81, 1.76)	1.62 (1.10, 2.46)
Log(Baseline PSA in ng/mL)	1.13 (0.76, 1.67)	0.77 (0.50, 1.19)
African American		
No		
Yes (ref)	0.40 (0.10, 1.28)	0.41 (0.09, 1.63)
Log(Gland volume in cc)	0.60 (0.30, 1.20)	0.94 (0.45, 1.96)
Clinical Failure Time	1.60 (0.60, 4.32)	1.60 (0.60 ,4.32)

© 2019 Beesley LJ et al. *JAMA Network Open.*

eTable 3. Posterior Means for Baseline Hazard Parameters in Model With Interactions

Parameter	Posterior Mean (95% CI)
$\log(\lambda_{12})$	4.87 (4.15, 5.72)
$\log(\lambda_{23})$	2.44 (1.55, 2.44)
ρ_{12}	1.09 (0.94, 1.24)
ρ_{23}	1.09 (0.91, 1.28)
b_1	0.005 (0.003, 0.008)
b_2	0.011 (0.006, 0.017)
b_3	0.026 (0.020, 0.043)
b_4	0.039 (0.026, 0.070)

© 2019 Beesley LJ et al. *JAMA Network Open.*

eTable 4. Estimated Treatment Hazard Ratio Across Model Specifications

Model	1->2	1->3	2->3	Overall Survival
Bayesian Illness-Death Model				
Main Covariate Set	0.80 (0.52, 1.23)	1.45 (1.12, 1.85)	1.59 (0.88, 2.68)	-
with Fewer Covariates*	1.04 (0.70, 1.90)	1.59 (1.24, 2.04)	1.64 (0.98, 2.65)	-
Cox Models for Overall Survival**				
Main Covariate Set	-	-	-	1.50 (0.97, 2.32)
with Fewer Covariates	-	-	-	1.50 (1.11, 2.01)
Unadjusted	-	-	-	3.45 (2.86, 4.16)
Cox Illness-Death Model**				
Main Covariate Set	0.98 (0.46, 2.08)	1.51 (0.94, 2.43)	1.50 (0.50, 4.50)	-
With Fewer Covariates	1.22 (0.75, 2.00)	1.42 (1.03, 1.97)	1.75 (0.91, 3.35)	-
Unadjusted Illness-Death Model***	2.37 (1.70, 3.29)	3.60 (2.92, 4.43)	1.84 (1.20, 2.83)	-

*Predictors: treatment, low/moderate/high risk group, Charlson score (0/1+), and age

**The adjusted Cox regressions use complete case data. For the Cox regression using the main covariate se we use N=2115 subjects. For the Cox regression with fewer covariates, we use N=3600 subjects. The Cox regression model fits use partial likelihood for estimation.

***Cox model fits adjusting only for treatment to the corresponding outcome.

© 2019 Beesley LJ et al. *JAMA Network Open.*

eFigure 1. Multistate Model Fit With Interactions

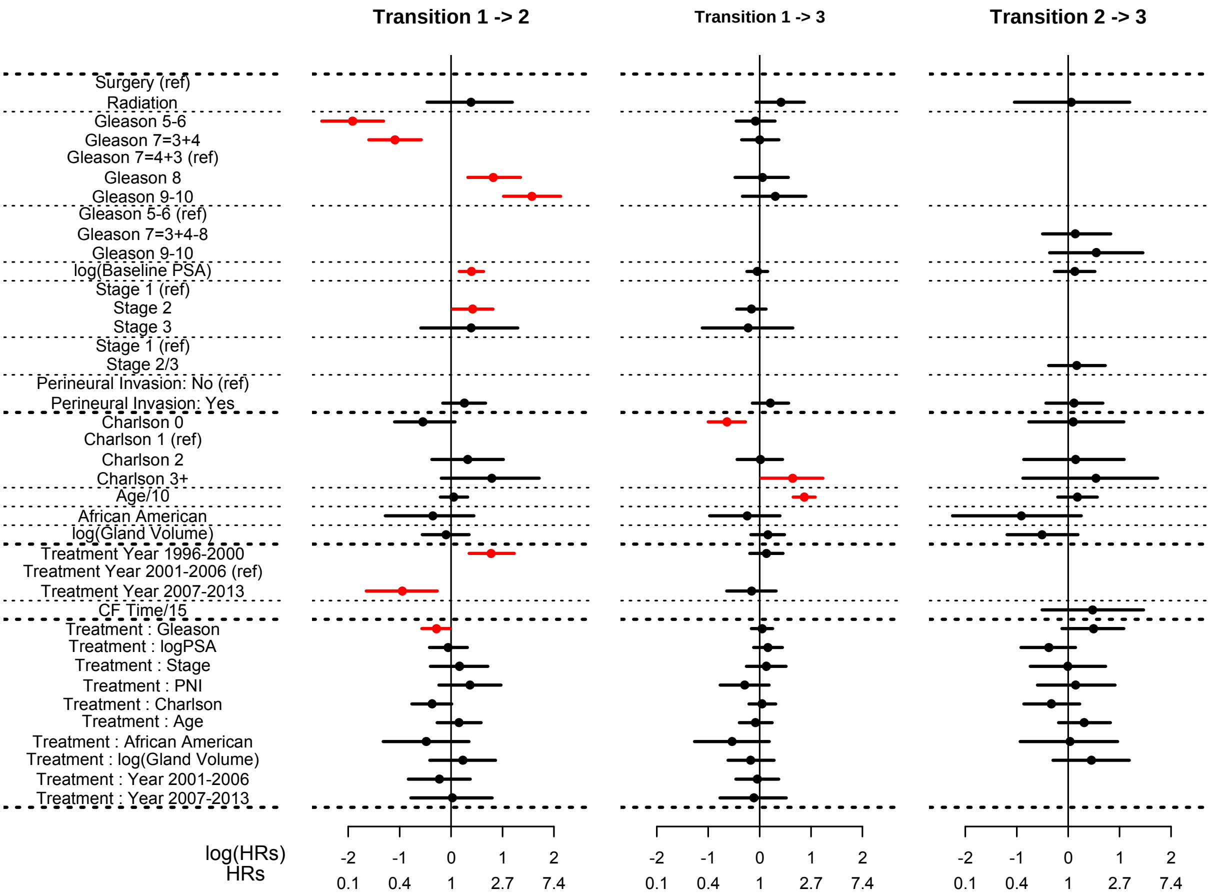

© 2019 Beesley LJ et al. *JAMA Network Open.*

eFigure 2. Estimated Treatment Associations by Patient Characteristics From Model With Interactions

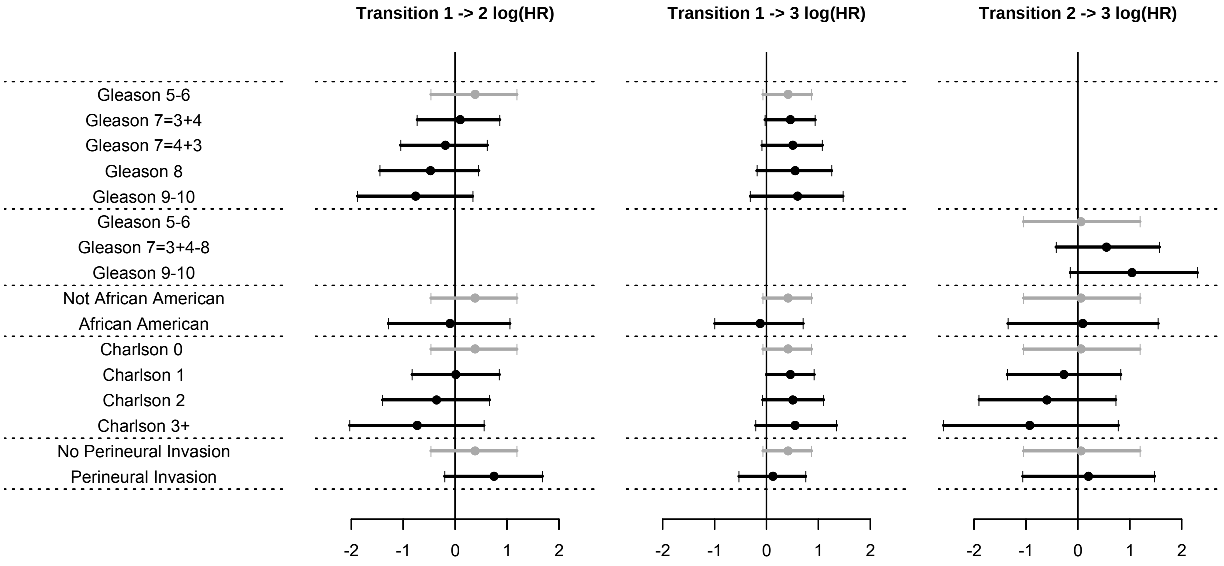

© 2019 Beesley LJ et al. *JAMA Network Open.*

eFigure 3. Predicted Probability of Clinical Failure/Death by 10 Years (N=2115, Complete Case Subjects)

(a) CF or Death

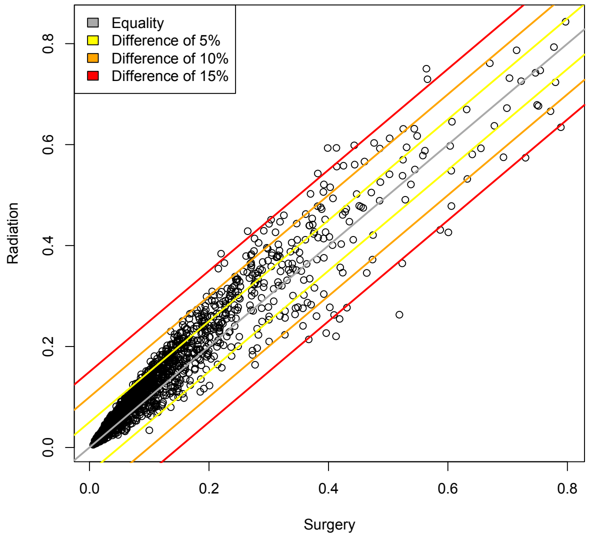

(b) CF

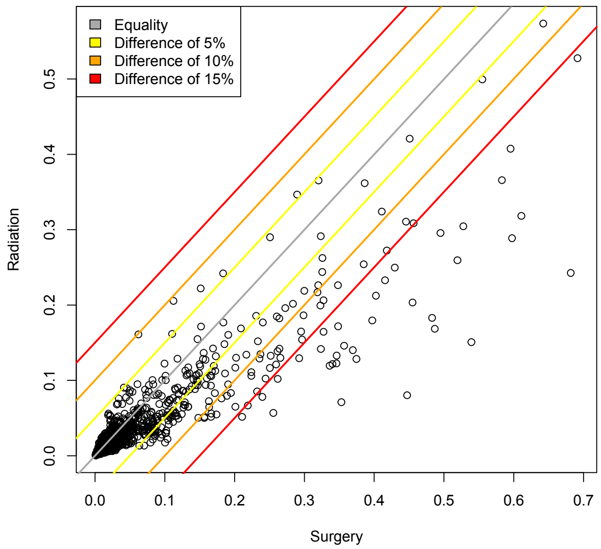

© 2019 Beesley LJ et al. *JAMA Network Open.*

eFigure 4. Predicted Probability of Clinical Failure/Death by 10 Years by Risk Group (N=2115, Complete Case Subjects)

(a) CF or Death

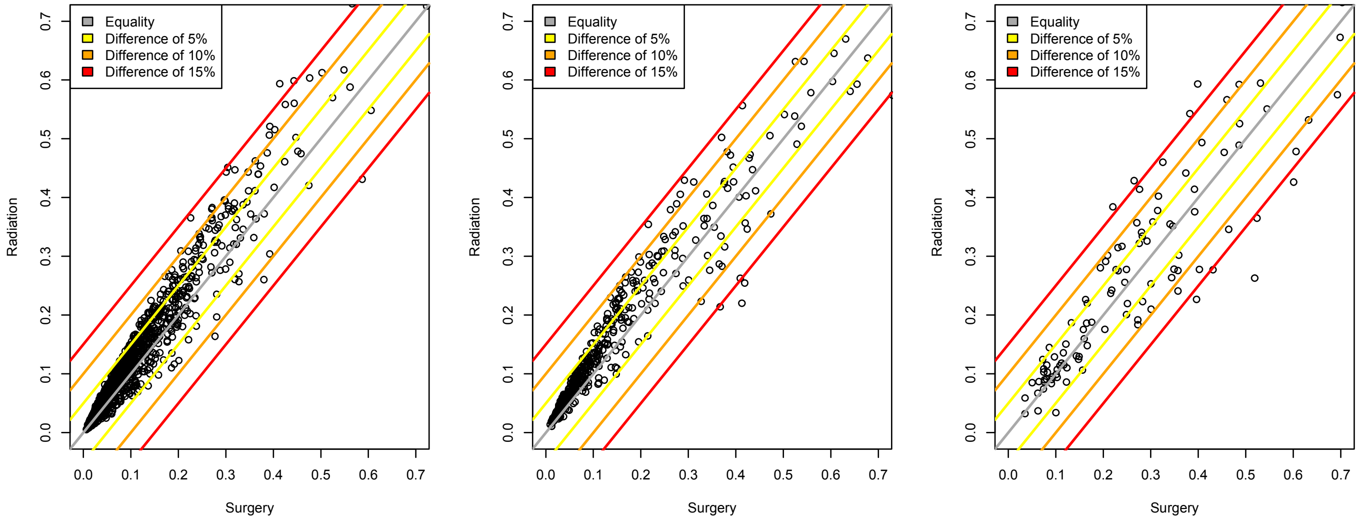

(b) CF

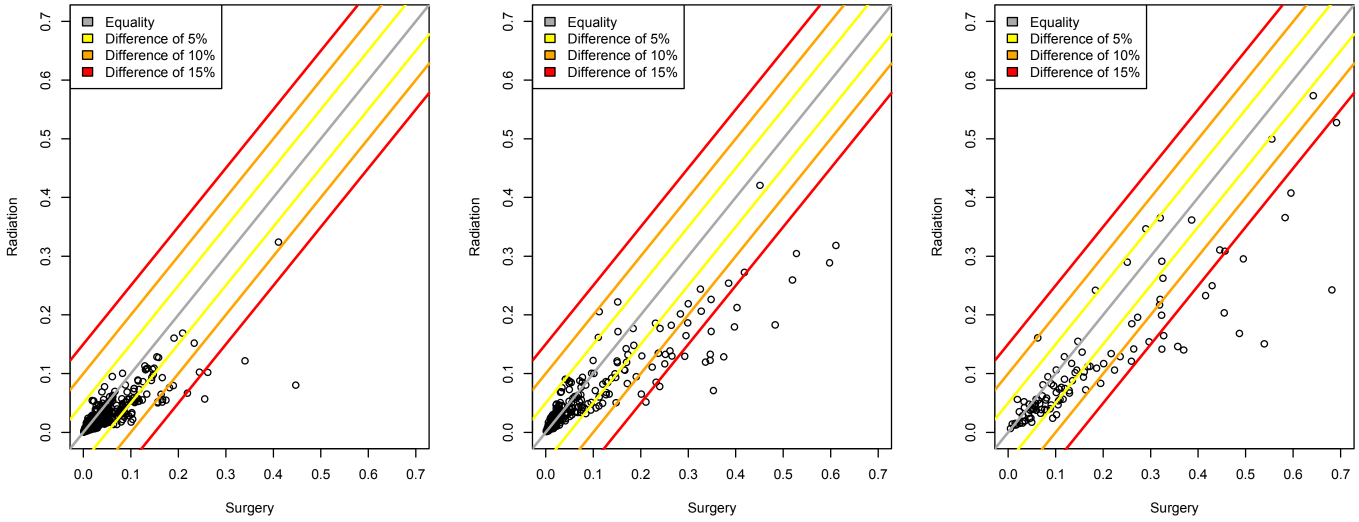

© 2019 Beesley LJ et al. *JAMA Network Open.*

eFigure 5. Predicted Probability of Outcome Events by 10 Years by Gleason Score (N=2115, Complete Case Subjects)

(c) CF or Death (d) CF

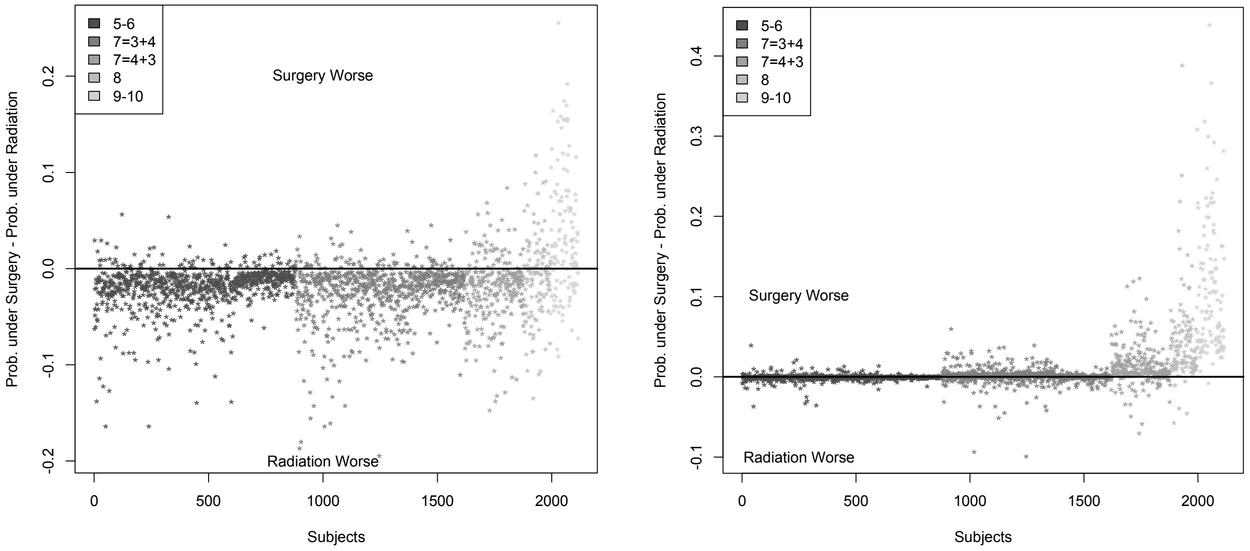

© 2019 Beesley LJ et al. *JAMA Network Open*.

eFigure 6. Bayesian Illness-Death Model With Fewer Covariates

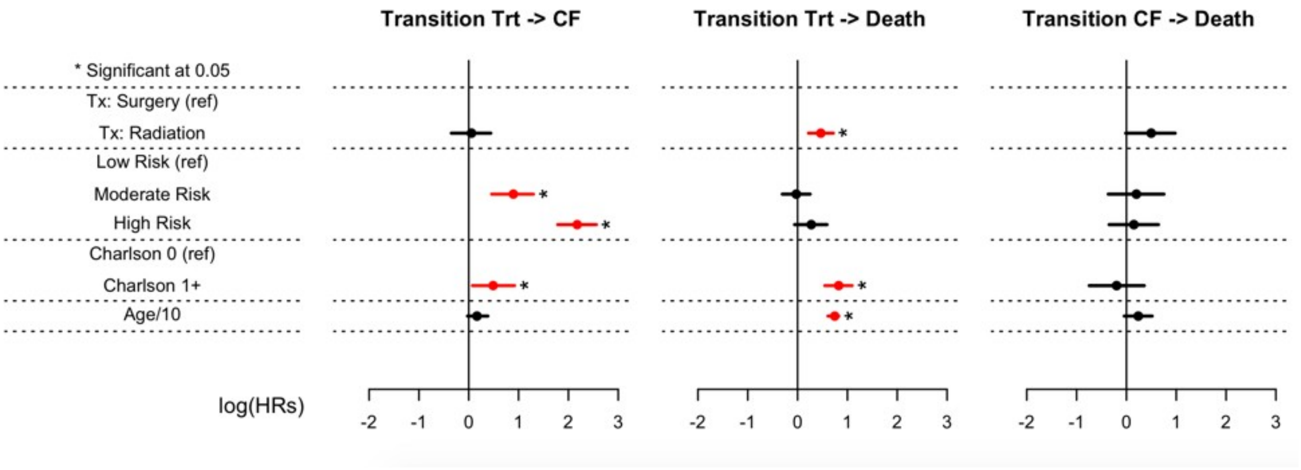

© 2019 Beesley LJ et al. *JAMA Network Open*.

eFigure 7. Theoretical and Estimated Associations of Treatment With Other Cause Death After Accounting for Different Degrees of Unmeasured Confounding*

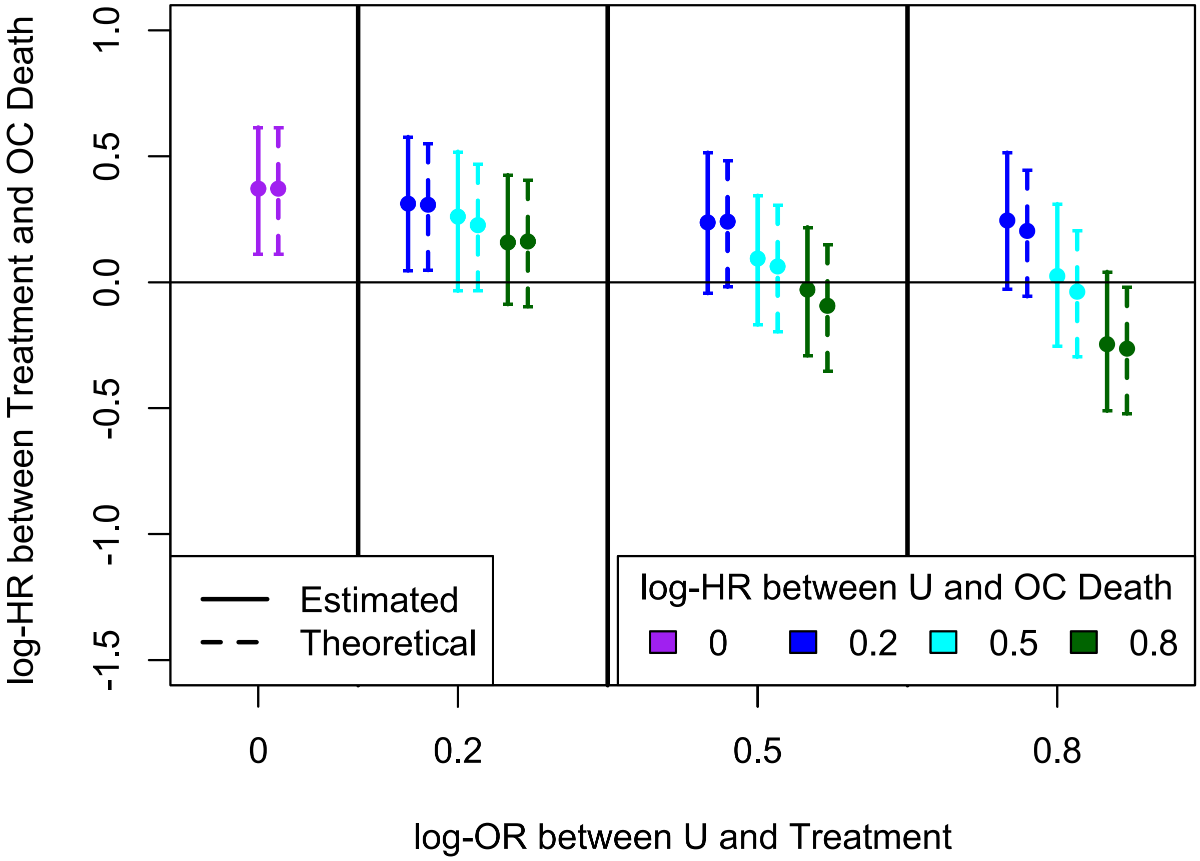

* Predicted log-HR obtained from equations presented in Ding and VanderWeele (2016).[4]

© 2019 Beesley LJ et al. *JAMA Network Open*.

eReferences

1. Bartlett, J. W., Seaman, S. R., White, I. R., Carpenter, J. R. & Alzheimer's Disease Neuroimaging, I. Multiple imputation of covariates by fully conditional specification: Accommodating the substantive model. *Stat Methods Med Res* **24,** 462–487 (2015).
2. Beesley, L. J., Bartlett, J. W., Wolf, G. T. & Taylor, J. M. Multiple imputation of missing covariates for the Cox proportional hazards cure model. *Stat Med* **35,** 4701–4717 (2016).
3. VanderWeele, T. J. & Ding, P. Sensitivity Analysis in Observational Research: Introducing the E-Value. *Ann. Intern. Med.* **167,** 268 (2017).
4. Ding, P. & Vanderweele, T. J. Sensitivity Analysis Without Assumptions. *Epidemiology* **27,** 368–377 (2016).

© 2019 Beesley LJ et al. *JAMA Network Open*.
